# Atmospheric oxidation and carbon contamination of silver and its effect on surface-enhanced Raman spectroscopy (SERS)

**DOI:** 10.1038/srep37192

**Published:** 2016-11-16

**Authors:** Antti Matikainen, Tarmo Nuutinen, Tommi Itkonen, Santtu Heinilehto, Jarkko Puustinen, Jussi Hiltunen, Jyrki Lappalainen, Pentti Karioja, Pasi Vahimaa

**Affiliations:** 1 Institute of Photonics, University of Eastern Finland, Joensuu, Finland; 2 Department of Environmental and Biological Sciences, University of Eastern Finland, Joensuu, Finland; 3 Center of Microscopy and Nanotechnology, University of Oulu, Oulu, Finland; 4 Faculty of Information Technology and Electrical Engineering, University of Oulu, Oulu, Finland; 5 VTT Technical Research Centre of Finland, Oulu, FI-90590 Finland

## Abstract

Surface-enhanced Raman spectroscopy (SERS) is considered a highly promising technology for different analytical purposes. The applications of SERS are still quite limited due its relatively poor quantitative repeatability and the fact that SERS is very sensitive to oxidation, which is a challenge especially with silver based SERS substrates. Here, the link between these phenomena is investigated by exposing silver SERS substrates to ambient laboratory air. We show that SERS intensity decreases exponentially after the exposure, which consequently leads to an increasing standard deviation (*σ*) in intensity. Within a five-hour measurement window, the SERS intensity already drops by 60%, while *σ* triples from 7% to 21%. The SERS results are supplemented by elemental analysis, which shows that oxidation and atmospheric carbon contamination coincide with the rapid SERS intensity decrease. The results emphasize how sensitive SERS is towards atmospheric contamination and how it can also reduce the measurement repeatability – even if the substrates are exposed to air just for a very short period of time.

Raman spectroscopy is a noninvasive and label free analytical method for characterizing material properties with light^1^. It is based on measuring Raman scattering, which is allows the chemical composition of the sample to be qualitatively and quantitatively analyzed. However, since Raman scattering of a photon is a rare event, Raman spectroscopy often requires high power excitation and high concentrations of substances. Its sensitivity can be improved by different techniques, such as resonance Raman (RR)^2^, coherent anti-stokes Raman (CARS)^3^ and surface-enhanced Raman spectroscopy (SERS)^4^, all of which aim to increase the probability of a Raman scattering event to occur through different measures.

In SERS, the signal enhancement mechanism is largely based on localized surface plasmons, which can confine light and its energy into a small volume and thus locally increase the excitation power. The plasmons are excited on metallic nanostructures, which can be fabricated in numerous different ways^5^,^6^ from different metals, but often silver is preferred due its suitable optical properties in the visible region i.e. its large scattering cross-section in comparison to the extinction cross-section^7^. In addition to this electromagnetic enhancement, the so called chemical enhancement mechanism brings about further enhancement when the analyte molecules form charge-transfer complexes with the metal surfaces^8^,^9^.

The enhancement in SERS is highly distance dependent. The electromagnetic enhancement is proportional to the fourth power of the excited plasmon field^10^, which in turn decays exponentially when the distance to the metal surface increases. In practice, this means that when the analyte is applied on the nanostructured metal surface (SERS substrate), only the molecules that are very close (<~3 nm^11^,^12^) to the surface, experience the field. Furthermore, the chemical enhancement occurs at even shorter effective range, as the molecules have to bond to the metal surface. Thus, even though high enhancement can be achieved by using SERS, the drawback is the enhancement and the resulting signal intensity tend to vary strongly.

Another issue with SERS nanostructures is the atmospheric corrosion of metals, including silver. When silver is exposed to air, it reacts with sulfur and oxygen species, which results in a gradual growth of silver sulfide^13^,^14^ or silver oxide^15^,^16^ layer. Although it is known that the corrosion influences the plasmonic properties of silver^17^, its effect on the SERS enhancement has been studied very little. Especially, when the SERS substrates are exposed to just ambient air. Furthermore, the speed at which the corrosion progresses and how fast its effect can be observed in SERS is not yet well known.

Here, we investigate how the SERS enhancement of silver based SERS substrates evolves after being exposed to ambient air. The signal enhancement as a function of air exposure time is studied with two different types of SERS substrates starting from the first minutes up to four days of exposure. The effect of air exposure on the elemental composition of the silver surface is studied using x-ray photoelectron spectroscopy (XPS). Finally, the effects of air exposure to the signal enhancement and measurement repeatability are discussed.

## Methods

The goal of this study was to inspect how exposure to ambient atmospheric conditions affects the SERS activity of silver based substrates. For this purpose, two types of SERS substrates were used: (i) silver nanoparticles (AgNPs) directly synthesized to solid surfaces and (ii) thermally evaporated silver films. The first one allowed us to monitor the temporal evolution of the SERS signal intensity in a minute scale, while the latter one also enabled the monitoring of chemical surface changes by XPS.

Rhodamine 6 G (Rh6G) was selected as the test molecule due to its popularity as a “standard” analyte in SERS research. Since the presence of Rh6G could change the course of oxidation and other surface processes, it was always deposited on the substrates after the air exposures.

### Preparation of AgNP SERS samples

**Figure 1:**
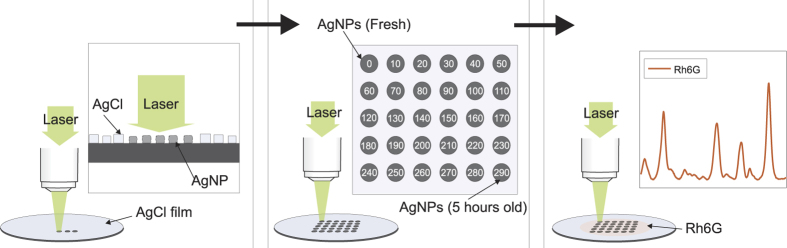
A schematic illustration of the 5-hour air exposure SERS experiment. A silicon wafer coated by a film of AgCl crystals was placed under a Raman microscope and a series of spots (30 in total) were exposed sequentially on the film at 10-minute intervals using laser light (*λ* = 514 nm). After exposing the last spot, a droplet of Rhodamine 6 G (Rg6G) was deposited on the wafer so that all spots and their silver were simultaneously covered. Thus, the first five hours of exposure to ambient air could be monitored by measuring the Raman spectra of Rh6G.

The AgNP’s were fabricated on a silicon wafer using the method described in ref. 18. The method is based on successive ionic layer adsorption and reaction (SILAR^19^), where insoluble salts can be grown in a cyclic, layer by layer process from their soluble precursors. Here, photosensitive silver chloride (AgCl) crystals were grown on a silicon wafer (Si-Mat, <100>) by sequential immersion to its precursor solutions: 5 mM NaCl (<99.8%, Sigma-Aldrich) and 5 mM AgNO3 (<99%, Sigma-Aldrich). After 50 cycles of immersions, the substrate was washed by incubating it for 5 minutes in ddH_2_O. In order to turn the formed AgCl crystals into metallic silver nanostructures, they were illuminated by high intensity laser light (Fig. 1). This approach allows a fast and precise sampling for short term air exposures.

Control over the air exposure time for the AgNP SERS substrate was achieved by exploiting the photosensitivity of the AgCl crystals. Small areas (*d* ~ 100 μm) of AgCl were turned to SERS active silver at a time by using the microscopes own laser (Fig. 1, left panel). For each spot a dose of (*P* = 5 mW, *t* = 5 min, *λ* = 514 nm) was applied. By using 5 minute breaks between the photoreductions, the sampling resulted in 30 “silver spots” representing the first five hours of exposure to air with 10 minute intervals (Fig. 1, middle panel).

For the SERS measurements, all spots were treated simultaneously; after 5 min incubation with 1 μM Rh6G in H_2_O, the surface was blown dry with compressed air (Fig. 1, right panel). The additional silver spots representing longer time points (24, 48, 72 and 96 h) were prepared in the same way.

### Preparation of evaporated silver films for SERS and XPS

The second type of SERS substrates was fabricated by the thermal evaporation of silver onto topographically patterned substrates. The surfaces were patterned in order to evoke SERS activity, which was done with the aid of polymer wrinkles (see ref. 20 for further information). Again, for the SERS measurements, the same Rh6G treatment was performed for each sample. For the XPS measurements, a film of 250 nm of silver was deposited on the surface of the silicon substrate so that the whole surface was fully covered and the native oxide layer of silicon could not interfere with the measurements.

### SERS measurements

All the SERS measurements were carried out right after the Rh6G treatment by using Renishaw inVia Raman microscope (*λ* = 514 nm) with a 5X objective, 50 μW power and 10 s accumulation time. With the AgNP substrates, the measurements were performed in the same order as the spots had been converted to metallic silver. With Ag films, the measurements were done with the same measurement parameters after 0, 24, 48, 72 and 96 hours after exposure to air. Each individual measurement point consisted of five parallel measurements and the obtained spectra were baseline corrected and then averaged.

### XPS measurements

In order to analyze the changes in the chemical composition of the silver surface caused by ambient atmosphere, the silver film sample was characterized with x-ray photoelectron spectroscopy (XPS) by using Thermo Fisher Scientific ESCALAB 250Xi XPS system. A monochromatic, Al Kα (1486.6 eV) x-ray source was used in the XPS measurements and the base pressure of the vacuum chamber was 10^−9^ mbar. Prior to the first measurement, the silver surface was cleaned with argon ion bombardment by using the ion gun of the XPS system to minimize the surface contamination. The XPS measurements were recorded after 0, 3, 6, 24, 48, 72 and 96 hours after initial exposure to air. Between the measurements, the sample was stored in a closed container in ambient conditions.

### SEM imaging

The scanning electron microscope (SEM) images were obtained with SEM Leo 1550 Gemini using 5 kV acceleration voltage.

## Results and Discussion

### SERS signal intensity as a function of air exposure time

**Figure 2:**
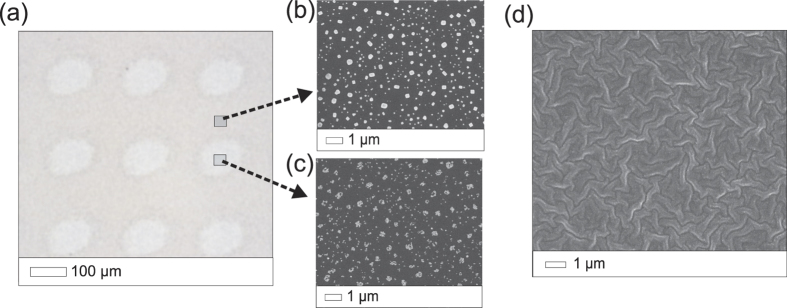
Optical microscope and scanning electron microscope (SEM) images of the two types of SERS substrates. (**a**) Optical microscope image showing the photoreduced spots of the AgNPs substrate. (**b**) SEM images showing the AgCl crystals and the (**c**) AgNPs that result from the photoreduction. (**d**) An SEM image of the evaporated silver SERS substrate.

The SERS enhancement was studied by measuring the Raman scattering intensity of Rh6G on two types of silver SERS surfaces: silver nanoparticles synthesized on a silicon wafer (AgNPs) and a film of evaporated silver (Ag film) (Fig. 2).

**Figure 3:**
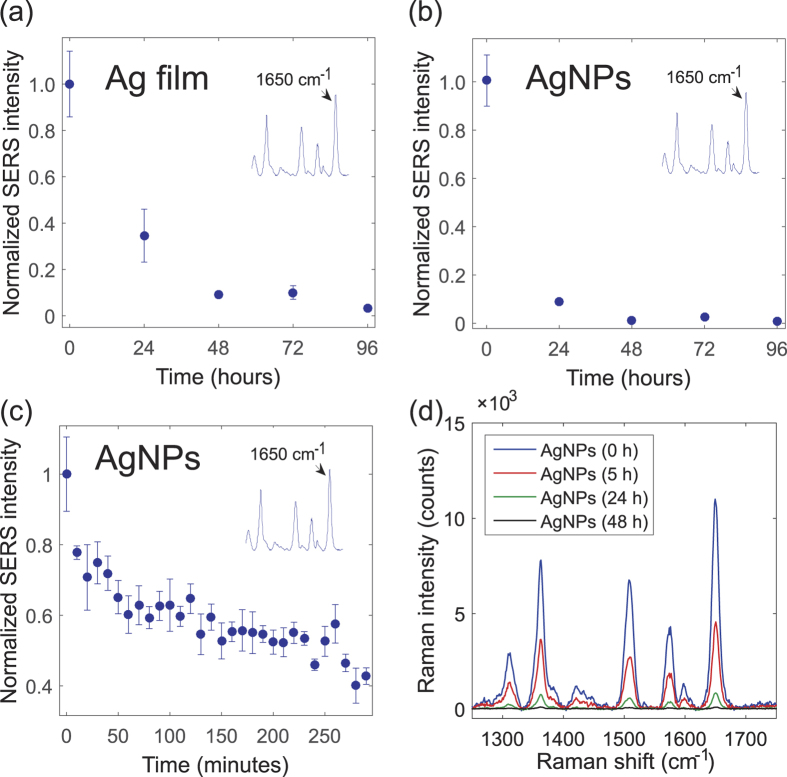
Surface-enhanced Raman spectroscopy (SERS) measurement results. Normalized SERS intensity of Rrhodamine 6 G as a function of air exposure time for the (**a**) evaporated silver (Ag film) and (**b**) silver nanoparticles (AgNPs). (**c**) Normalized SERS intensity of AgNPs for the first five hours of exposure to air. (**d**) Raman spectra of Rh6G measured on fresh and 5, 24 and 48 hours old silver. The inset in (**a**–**c**) shows an example Raman spectra of Rh6G and the peak at 1650 cm^−1^, which is used as an indicator for the SERS intensity. Each measurements point is an average of 5 measurements and the error bars correspond to the standard deviation (*σ*) of each measurement.

For both sample types the SERS intensity was first measured at one day intervals for the duration of four days. The effect of air exposure can be immediately seen as a rapidly decreasing SERS signal intensity (Fig. 3). The decay seems to be slightly faster with the AgNPs than with the evaporated silver, which may be due to the larger surface-to-volume ratio of the AgNPs. The decay is nonetheless exponential with both sample types. With the AgNPs, only ~10%, and with the evaporated silver, only ~30% of the original signal intensity could be obtained after one day of exposure to ambient air. After two days, the signal was practically completely lost with both sample types. Somewhat similar results have been previously reported with silver based SERS substrates that were exposed to sunlight and environmental ozone^21^. Here however the substrates were stored in the dark, which suggests that the phenomenon exists also without the oxidation promoting treatment.

We also wanted to test how the SERS intensity decreases within a “realistic” SERS measurement session, which lasts for a few hours after the substrates have been exposed to air. For this purpose, the first five hour of exposure were monitored with shorter, 10-minute intervals. The measurements recorded during this period revealed that a lot of the signal decay actually took place already within the first minutes (Fig. 3c). In fact, with the AgNPs the signal intensity dropped by 20% just after 10 minutes of exposure to air. After that the SERS intensity continued to decrease more moderately, but at the 5-hour mark it had already reduced by 60% in comparison to the enhancement obtained with fresh silver.

It is noteworthy that this kind of rapid decay does not only affect the magnitude of the enhancement but it also significantly affects the measurement repeatability. The measurements were individually quite repeatable, as the standard deviation (*σ*) was only ±7% on average for 30 time points. However, if all measurements taken during the 5-hour period are considered as a whole, the standard deviation triples to σ = ±21% due to the constantly reducing signal intensity. Thus, when the SERS measurements extend over several hours – as they often do – notable signal intensity variation can be expected solely due to this effect.

### The origin of the SERS enhancement reduction

The results of the SERS experiments are rather indirect observations in the sense that they do not reveal what exactly causes the signal intensity decrease. To shed light on the causes of the enhancement drop, the surface of the silver sample was characterized by using XPS.

**Figure 4:**
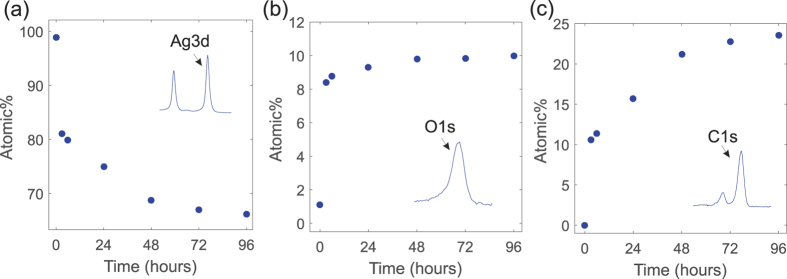
X-ray photoelectron (XPS) measurement results recorded 0, 3, 6, 24, 48, 72 and 96 h after exposure to ambient air. The calculated relative atomic percentage (Atomic%) of (**a**) silver (**b**) oxygen and (**c**) carbon on the silver substrate surface as a function of air exposure time. Atomic% was calculated from the Ag3d, O1s and C1s peaks for silver, oxygen and carbon respectively.

The XPS data shows that the elemental composition of the silver surface changed remarkably during the four days of exposure (Fig. 4). The relative amount of detected silver decreased rapidly due to the increasing percentage of oxygen and carbon species on the surface. After four days, the elemental composition of the SERS substrate surface had changed from nearly pure silver (99% silver, 1% oxygen) to 66% silver, 10% oxygen and 24% carbon.

In addition to oxygen and carbon, also small amounts (0.33%) of sulfur were detected but only after the samples had been exposed to air for at least two days. This is somewhat surprising, as silver sulfide formation is generally considered one of the main causes of the corrosion in silver^13^,^14^,^17^. This may be simply due to the fact that the composition of “ambient air” varies slightly from a location to another and in these experiments the sulfur compounds were just mostly absent. Thus, in these experiments the sulfur contamination did not seem to contribute – at least directly – to the SERS enhancement decrease.

However, in terms of oxygen and carbon contamination the XPS results also seem to be in good agreement with those of SERS. The rapid SERS enhancement decrease coincides with the rapid rise of the amount of oxygen and carbon, which consequently leads to the reduction in the amount of silver detected. Because the samples were cleaned with the argon ion bombardment before the first measurement, both oxygen and carbon must have been originated from the ambient atmosphere itself. The carbon contamination is likely caused by the adsorption of hydrocarbons from the surrounding atmosphere leading to the formation of a thin carbon layer on surfaces^22^. The origin of the oxygen peak cannot be explicitly deduced from the data, but it is likely a combination of chemisorbed oxygen species, native oxide layer and oxygen containing carbon species adhered on the silver surface^23^.

Thus, the presence of oxygen on the silver surface can affect SERS in many ways. If the O1s peak originates at least partly from a silver oxide layer, the SERS signal intensity decrease could be attributed to plasmon damping^17^ and the fact that the oxide layer simply obscures the metal surface and prevents the Rh6G molecules from reaching the high enhancement areas. It is also possible that the enhancement is partly reduced due to chemisorbed oxygen^21^, which can disrupt the binding of the analyte molecules and weaken the chemical enhancement contribution.

Interestingly, the SERS intensity still kept decreasing, even though the presumed oxidation had already stabilized (Figs 3 and 4). Our data implies that this further signal decrease could be due to the carbon contamination, which continued to build up for several days after the amount of oxygen no longer increased. Also, it seems to correlate strongly with the SERS intensity decrease. It is known that carbon contamination makes metal surfaces more hydrophobic^24^, which suggests that the increase in the carbon content may at least affect the binding of the Rh6G molecules and weaken the chemical enhancement mechanism. Furthermore, carbon contaminates can increase the measurement background noise^25^, which can also have a negative effect on the observed signal enhancement. In summary, the results indicate that its impact on SERS may be more significant than generally expected.

In conclusion, the present results show that both the oxidation and the carbon contamination affect the signal enhancement and measurement repeatability significantly even when the exposure time to the ambient air is very short. Furthermore, as the carbon build-up is not a property of silver alone; the results implicate that it could be an issue with other metals used in SERS, like gold, which is otherwise more resistant to the oxidation. The carbon contamination might also be a problem in other plasmonic detection schemes, such as plasmon-enhanced fluorescence spectroscopy (PEF)^26^. Thus, the results highlight the sensitivity of metal surfaces towards atmospheric contamination, which is – in our opinion – an issue that should not be overlooked in SERS or other plasmonic applications.

## Additional Information

**How to cite this article**: Matikainen, A. *et al.* Atmospheric oxidation and carbon contamination of silver and its effect on surface-enhanced Raman spectroscopy (SERS). *Sci. Rep.* **6**, 37192; doi: 10.1038/srep37192 (2016).

**Publisher’s note**: Springer Nature remains neutral with regard to jurisdictional claims in published maps and institutional affiliations.
